# Mode of Treating Itch at Berlin

**Published:** 1843-03

**Authors:** 


*Mode of Treating Itch at Berlin.*—By Dr. Hauck.—The treatment of itch has lately been made the subject of extensive experimental observation in the Berlin hospitals, and it is satisfactory to learn that a slight modification of what is termed the English method, has been found in every respect superior to any other that was adopted, accomplishing as it does all the desirable objects of curing the disease quickly, certainly, and economically.

The remedy employed was the sulphur and soap liniment of the Prussian Military Pharmacopœia, composed of one part of flowers of sulphur, and two parts of soap mixed with sufficient hot water to make them into a soft ointment. The patients, after a warm bath of soap and water had been applied, were placed undressed in a chamber, kept constantly at a temperature of 95° F., and well rubbed with the ointment over all the parts where the eruption had appeared, three times a day, and then made to sweat profusely by putting them in warm beds. This system was continued for three days and nights; on the morning of the fourth each patient had a warm bath, and then if not cured, was provided with clean bed and body-linen, and put in a ward of ordinary temperature, while the suspicious parts were still rubbed with the ointment, and a warm bath taken every other day. In general, no external medicines are given; but the diet allowed was reduced to a fourth portion, and water only given to drink.

In this manner, but with one short interval, 1981 were treated and cured between September, 1839, and February, 1840, making the total number of days of treatment 15,890, which gives on an average eight days and a small fraction for the cure of each of patient, and for the expense of each about two dollars. The exact result was, that—in three days there were cured 42, in four days 161, in five days 333, in six days 376, in seven days 207, and in more than seven days 859.

The treatment of these last was prolonged by many circumstances which can hardly cast discredit on the remedies. In many among them the itch was soon cured, but they remained under treatment for the ulcers which come on from long neglect of it, or were kept in the hospital till there was no chance of the ulcers communicating the disease. Others among them after being cured of the skin disease had to be treated for other affections, such as ophthalmia, fever, &c.; and others again had their cure delayed by an obstinate refusal to adopt all accessory treatment. And in addition to these causes giving rise to an apparent increase of the length of time necessary for the cure of the disease, there were some others dependent on the management of the hospitals, and other circumstances quite foreign to the treatment adopted, but which, had they not existed, would have permitted the average number of days of treatment to have been stated much lower.

In the whole fifteen months there occurred only eight cases of relapse, less that is than half per cent. of the cases treated; and among these there was, in many, good reason to suspect a fresh infection. The other cases in which there appeared to be a relapse were in fact only examples of eczema resulting from the stimulus of the skin by the sulphur. In no case did the treatment of the skin give rise to any general disorder, or to the inflammations and congestions which some have described as resulting from it.—*N. Y. Lan.*, from *British and Foreign Medical Review.*

